# Independent Professional Medical Journals Are More Peculiarly Representative of the Profession Than Are Society Organs

**Published:** 1907-05

**Authors:** 


Independent Professional Medical Journals Are More Peculiarly Representative of the Profession Than Are Society Organs.—Any attack upon their existence by organized power is a direct blow at the profession itself. To them, more than any other agency, has been due the development of the movement toward solidarity, as well as the advance of science or reform. The initiative has always been theirs, and, so long as progress springs from the work of the few instead of from the work of organized bodies, they will be needed that growth may come. They alone can represent independent thought, or exercise the wholesome function of disinterested criticism. Yet under present conditions, it is in the power of a few men in the Association, by employing the competitive methods of a monopoly, to make the existence of independent journals impossible. These journals may be threatened with the boycott unless they are entirely subservient to those controlling the Association. They may be crowded out by unfair competition in the advertising field, or by pressure exerted in various ways; for example: By the Association and State journals adopting rates proportionately so low that journals unsupported by organization fees will be unable to compete; by using the organization as a club, "blackmailing" the advertiser in a cunning way to secure patronage for the organization journal and bar out a rival; by the organization journals taking advertising until proved unfit, and claiming the right of such proof under control so that none may say conclusively that exclusion and ability to dispense with such revenue are coincident. Unfair competition may also exist in the matter of literary and scientific material. Efforts may be used to secure for organization journals literary and scientific material not constructively nor in any sense justly to be claimed by them. Yet these journals are frequently unable to publish fully their own transactions for "lack of space!" Authors may be shown that unless their articles are published in these organization journals they will, so far as possible, be denied quotation and reference in their pages. It is publicly stated that all these methods have been employed in concerted attack, both open and secret, upon independent medical journals of high professional standing.

TRADES-UNION METHODS are not in the spirit of the profession. They have been initiated and carried out by the few for self-aggrandizement directly, or indirectly by means of the accumulation of a large surplus. They will ultimately be condemned by the great body of the profession, but this should be done while prevention of ill-effects is still possible. It is unseemly that organization journals should carry any advertising which they may be compelled at any time to exclude or denounce. Nor should the organs of professional scientific bodies act as the mouth-piece of manufacturing or publishing firms. Higher ethics would dictate that they devote themselves to organization and professional matters, and publish no advertising. Particularly is this true if reform in advertising is to be carried out through their agency, or by their attack. General confidence in the purity of their motives will never be felt so long as present methods prevail. President Bryant speaks of these matters in no uncertain terms: "Whether or not the medical journals which, for covetous business reasons, exploit remedial agents in a manner calculated to deceive the unwary, the unsophisticated and the indolent, are to receive professional support, will, as it seems to me, be determined more by the outcome of patiently developed, and higher comprehensive professional sense, than by other inhibiting influences."

REFORMS DEMANDED IN THE INTERESTS OF PROGRESS.—Briefly these may be summarized:

1. Verbatim reports of the proceedings of legislative and governing bodies.
2. Itemization and utmost publicity of financial matters.
3. Proper representation in the offices of secretary, of editor, and of business manager by separate individuals, with proper compensation.
4. Nondiscretionary power of the editor, with government by the sections of the published proceedings.
5. The rendering impossible of trades unionism and monopolistic methods.
6. Provision for general secret ballots upon important questions of policy by means of the machinery of the Association and its journal, through district and county societies.
7. The extension of the referendum and initiative from the optional legislative to the popular and obligatory form.
8. In order to protect apparent minorities, placing the vote necessary for both referendum or initiative upon a reasonable basis.
9. The rights of individual members must be held inviolate from attack by those in power.
10. The Association and its journal must be enjoined from entering into purely commercial competition to the detriment of its professional rivals.
11. No paid agent of the Association should be permitted to be a member of, or take part in the deliberations of, the bodies governing or directing his actions or compensation.

These reforms are demanded by a large and increasing body of the profession, among whom are many members of the Association, and not a few of its officers. They are here presented for discussion by the House of Delegates at the approaching annual meeting. May that body have wisdom sufficient for reform from within lest reform be forced from without.—*Advance sheets edi-*

*torial pages American Medicine for May. Dr. Geo. M. Gould, Editor.*

